# Supplementary figures and images for: Delayed evoked potentials in zebra finch (Taeniopygia guttata) under midazolam-butorphanol-isoflurane anesthesia
Source: PeerJ. 2019 Oct 24;7:e7937. doi: 10.7717/peerj.7937 (PMC6815651; doi:10.7717/peerj.7937)

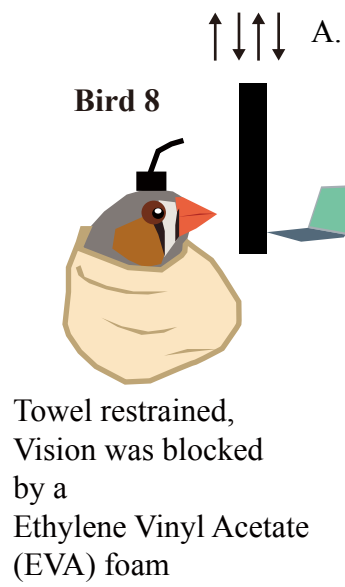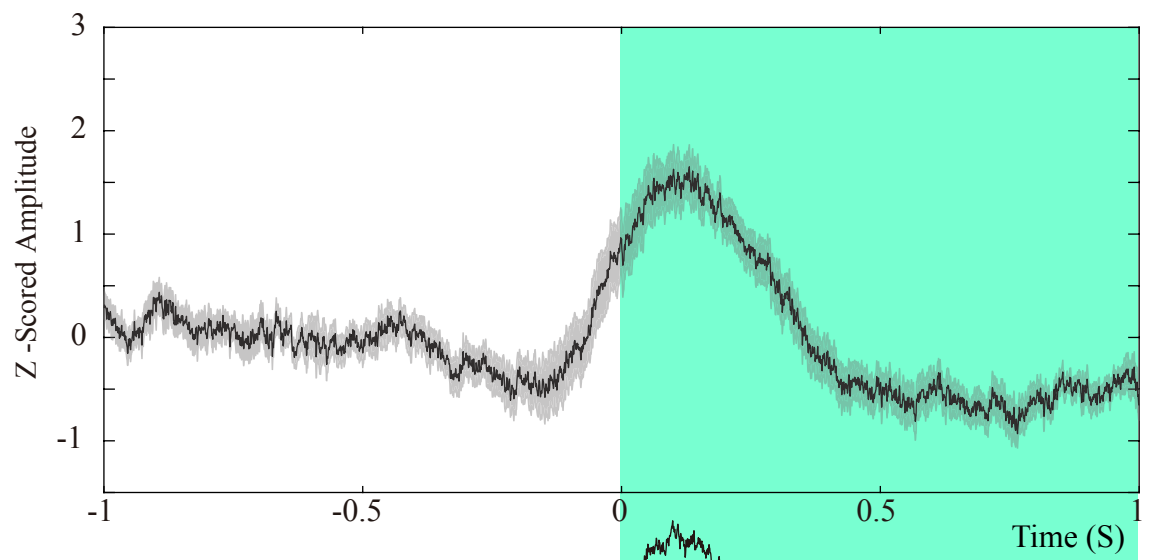

B.

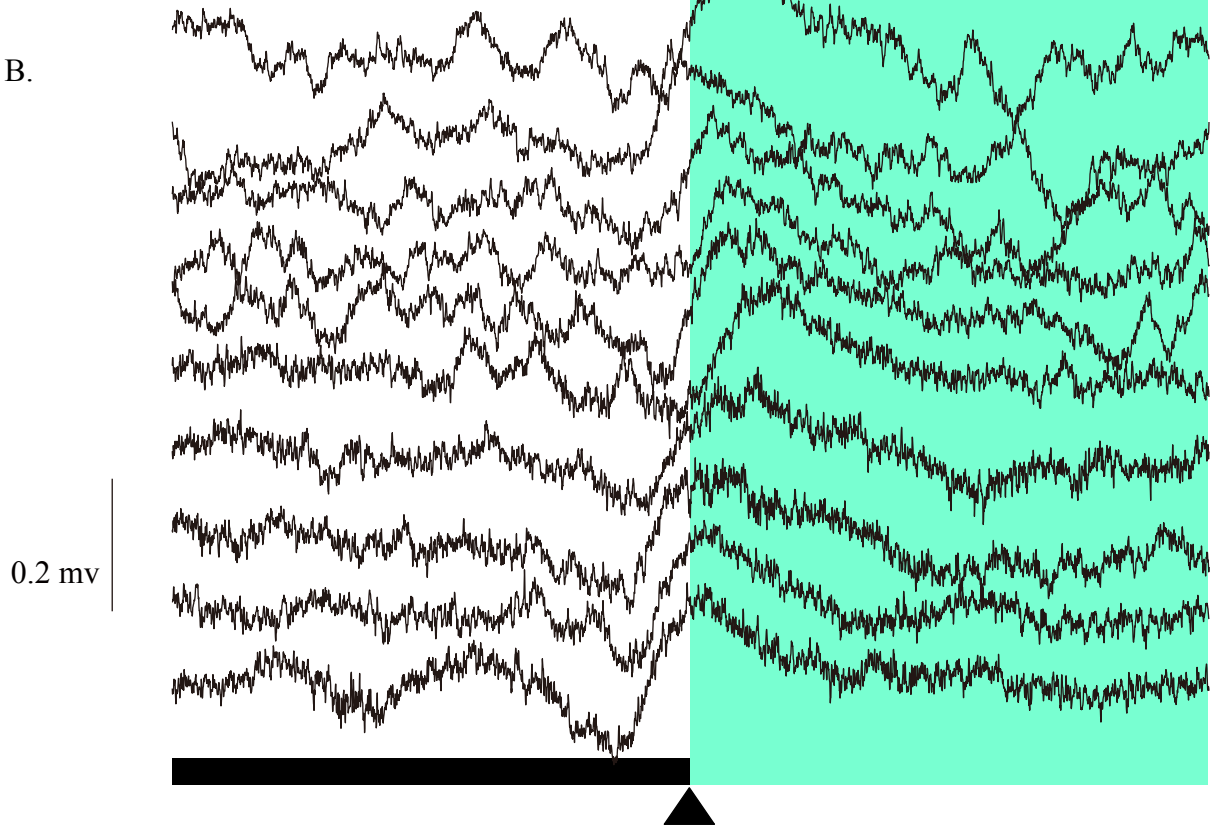

C.

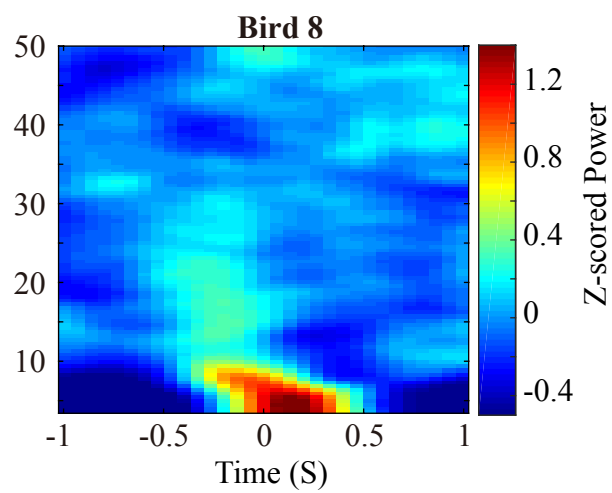

Supplement: Supplemental Information 1 — (A) Averaged LFP traces. (B) Ten examples of raw LFP traces. The triangle indicates the time when the foam was elevated. (C) An averaged spectrogram. [file peerj-07-7937-s001.pdf]

A.

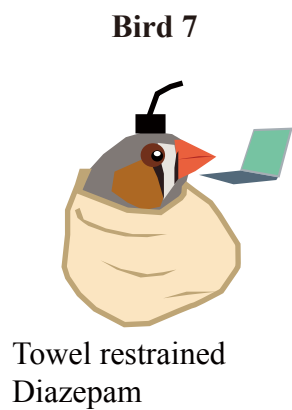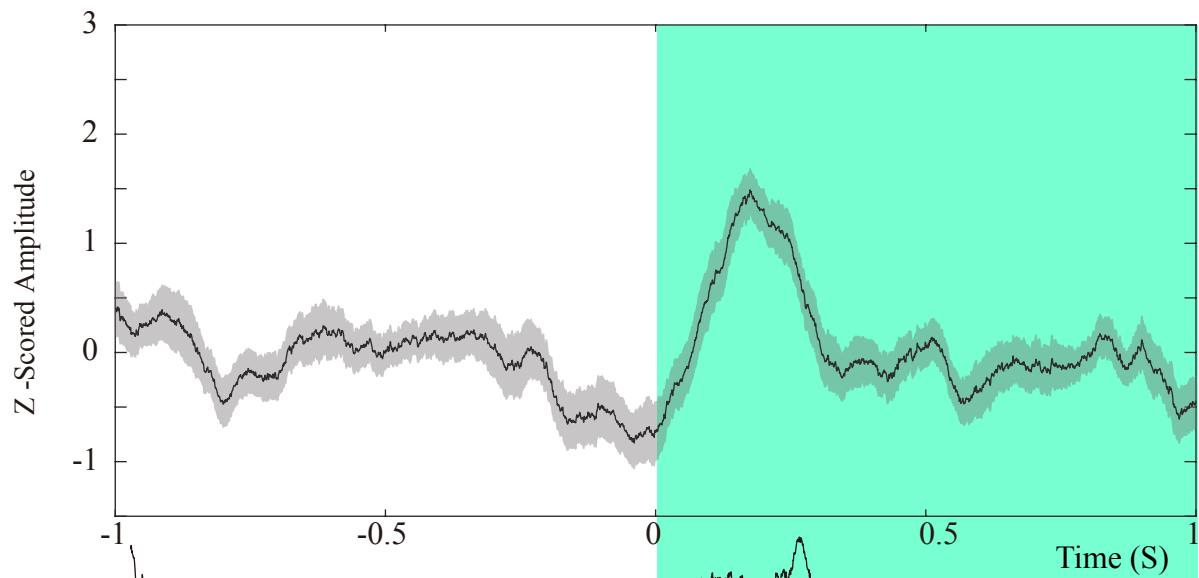

B.

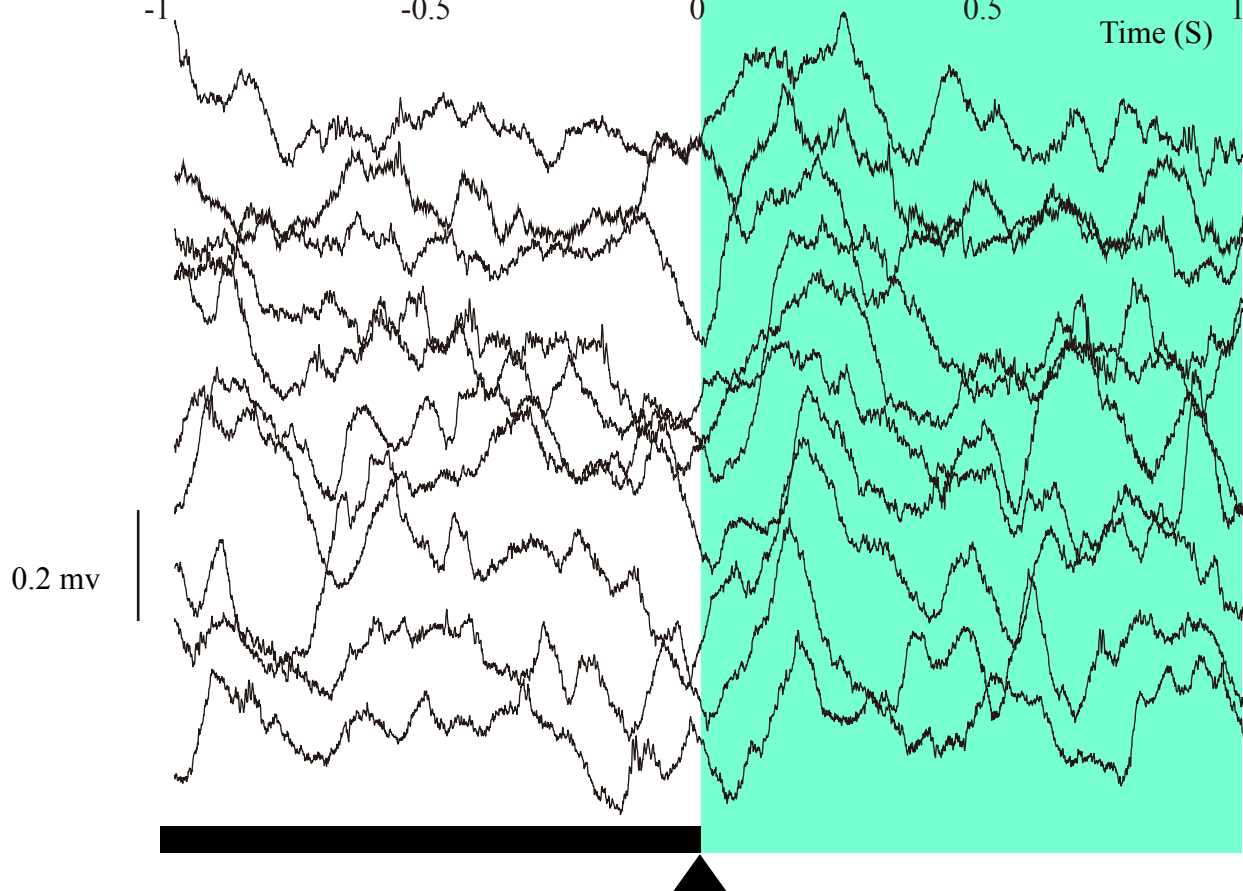

C.

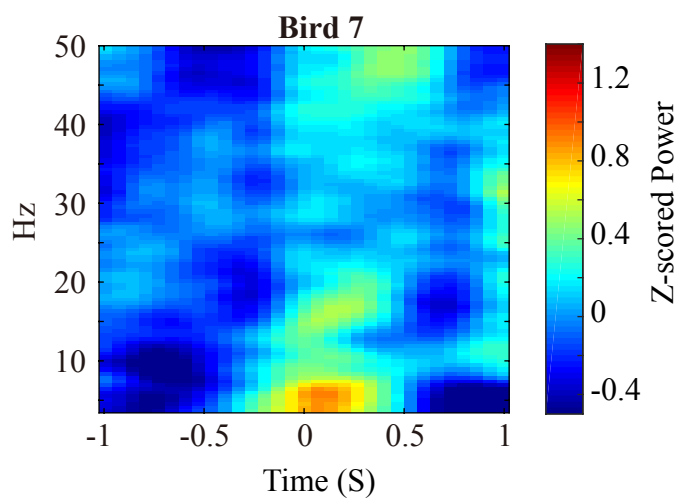

Supplement: Supplemental Information 2 — (A) Averaged LFP traces. (B) Ten examples of raw LFP traces. The triangle indicates the time of stimulation. (C) An averaged spectrogram. [file peerj-07-7937-s002.pdf]

# Bird 5 (control)

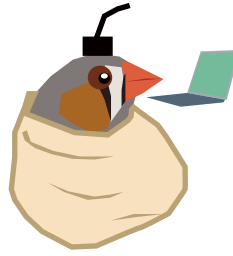

Towel restrained

Raw LFPs

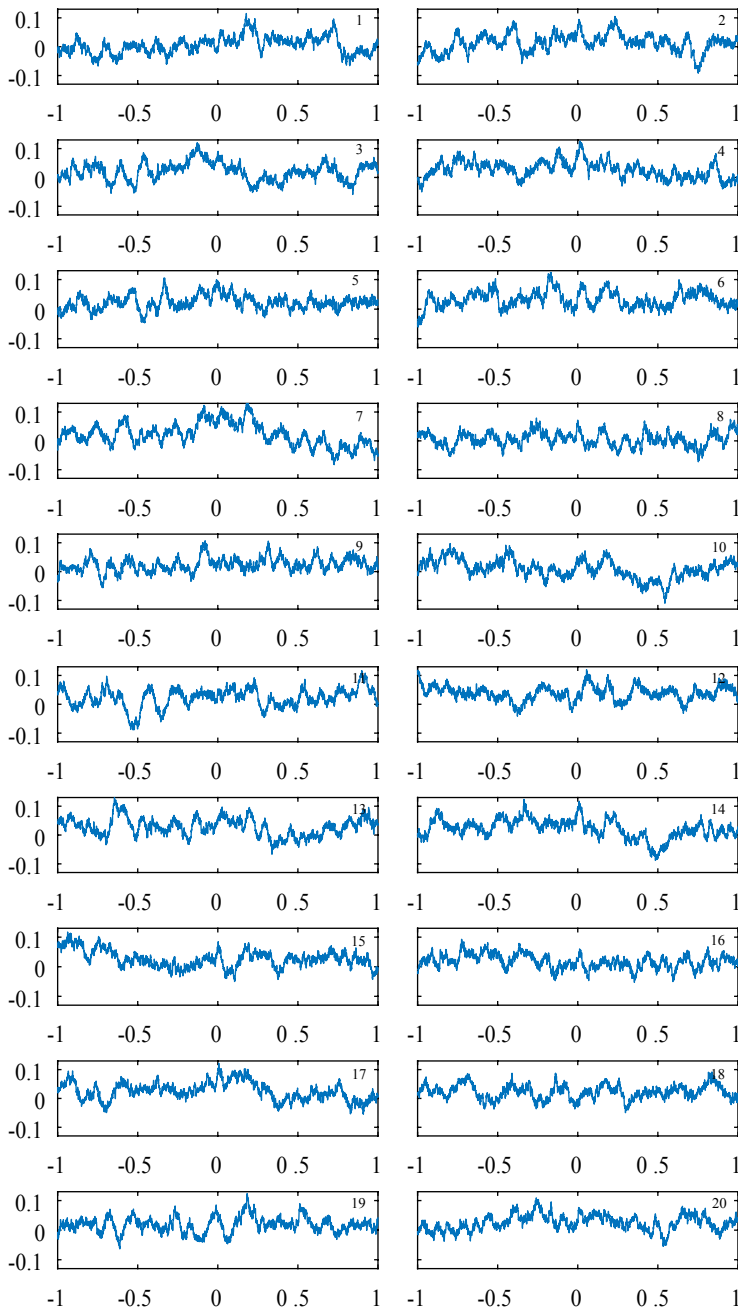

ICA processed LFPs

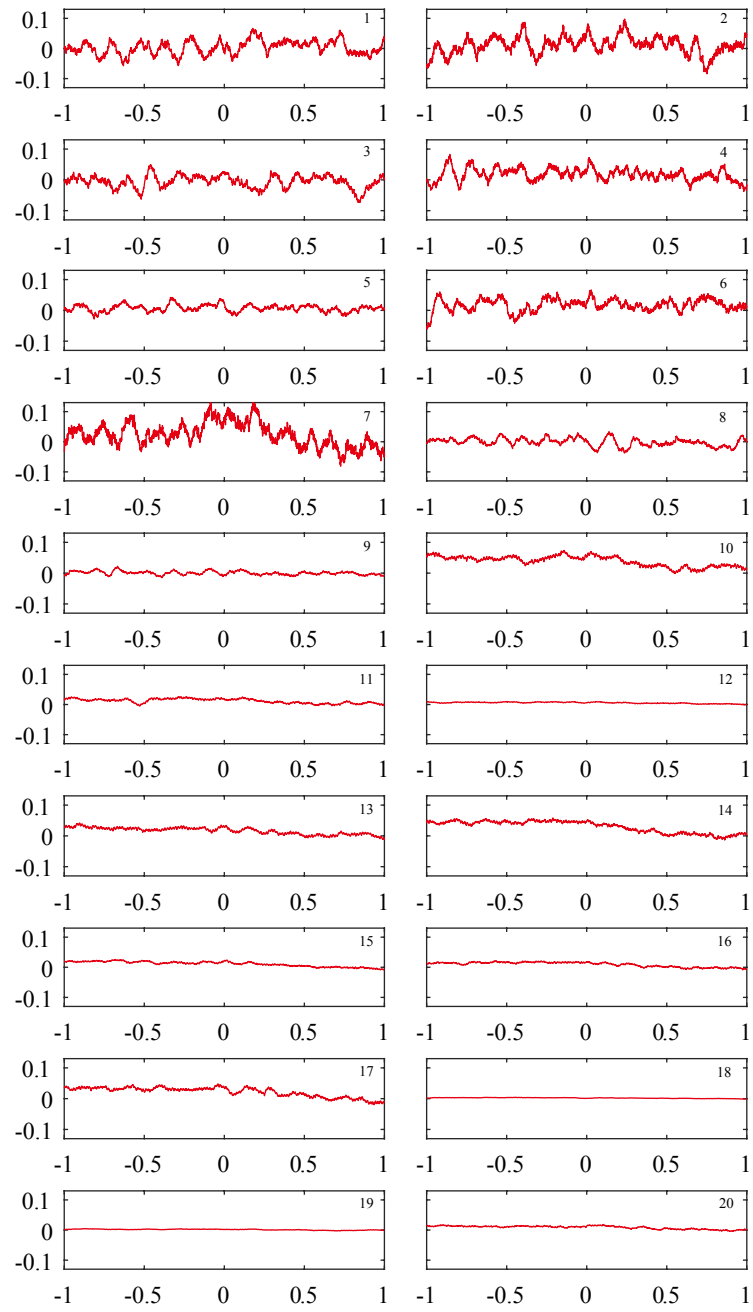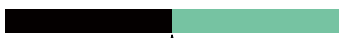

Time (s)

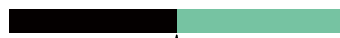

Time (s)

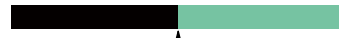

Time (s)

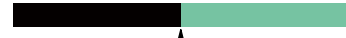

Time (s)

Supplement: Supplemental Information 3 — Blue traces are raw traces (units on Y axes: mV). Red traces were pre-processed by ICA (units on Y axes: mV) for denoising. The numbers beside each trace represent the orders of the trials. [file peerj-07-7937-s003.pdf]

# Bird 6 (control)

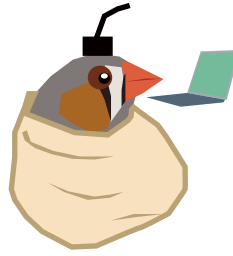

Towel restrained

Raw LFPs

ICA processed LFPs

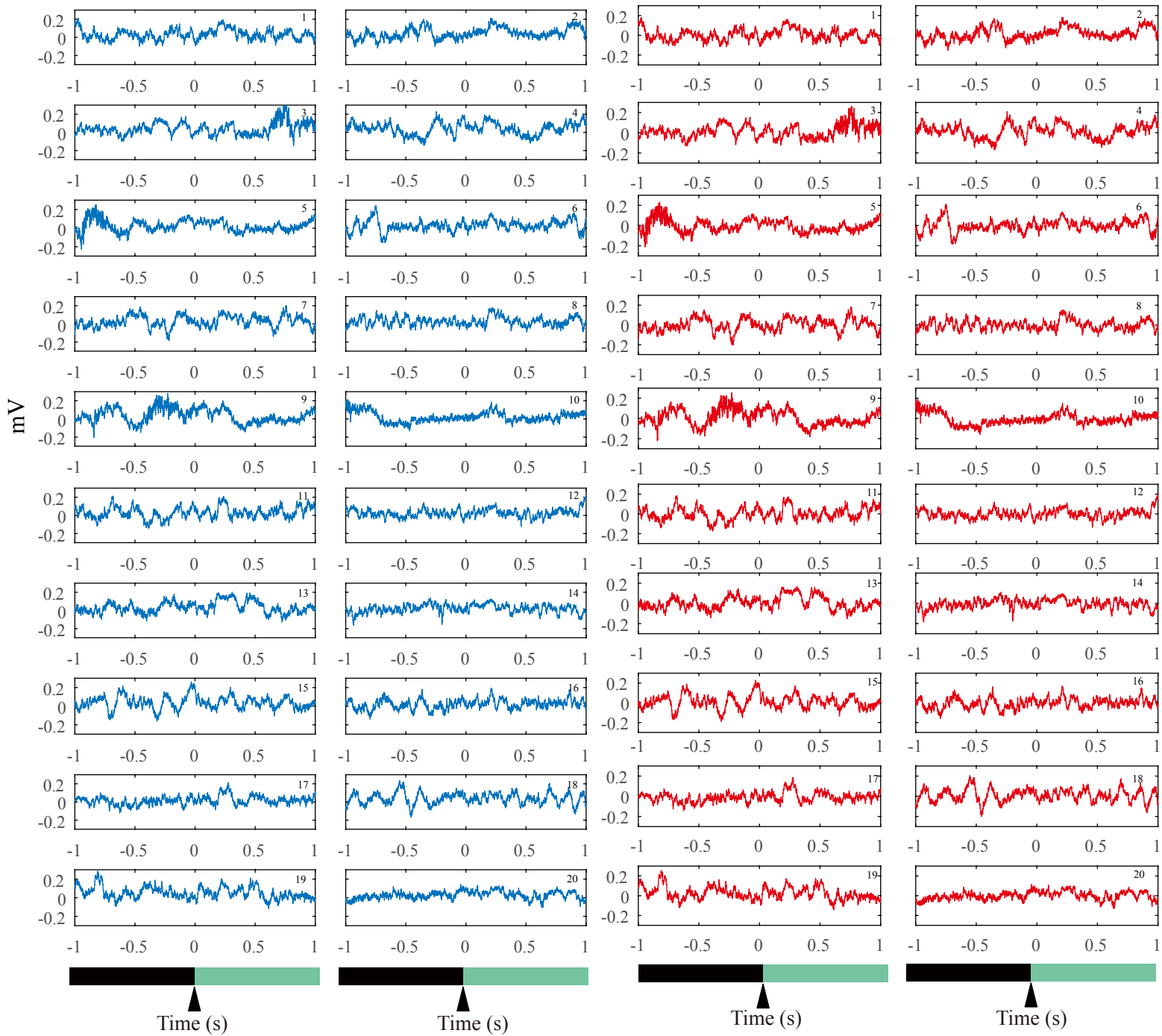

Supplement: Supplemental Information 4 — Blue traces are raw traces (units on Y axes: mV). Red traces were pre-processed by ICA (units on Y axes: mV) for denoising. The numbers beside each trace represent the orders of the trials. [file peerj-07-7937-s004.pdf]

**Bird 5 (control)**

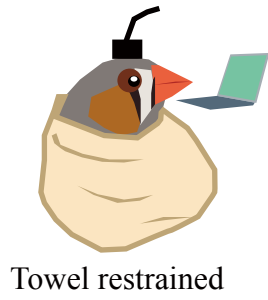

A

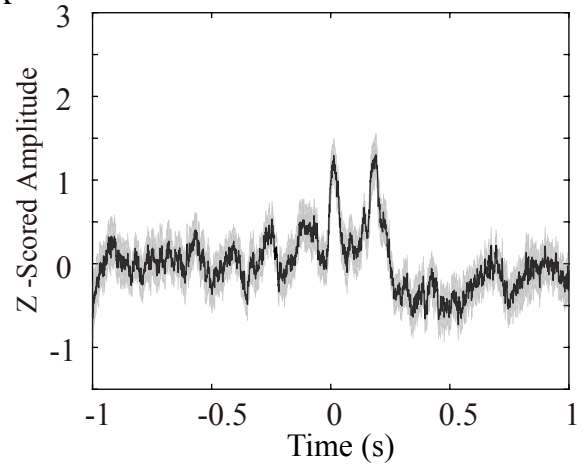

B

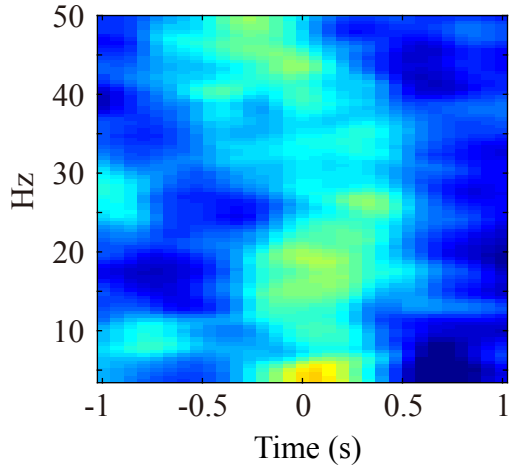

C

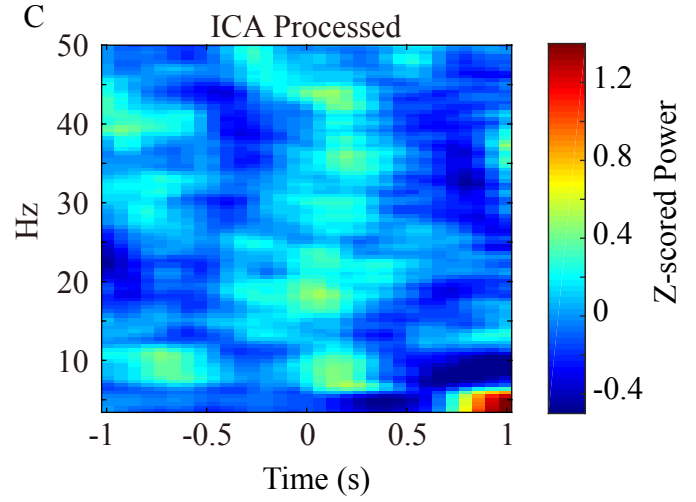

**Bird 6 (control)**

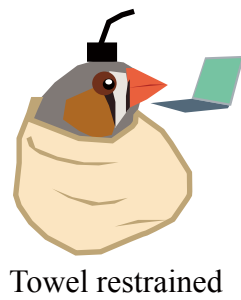

D

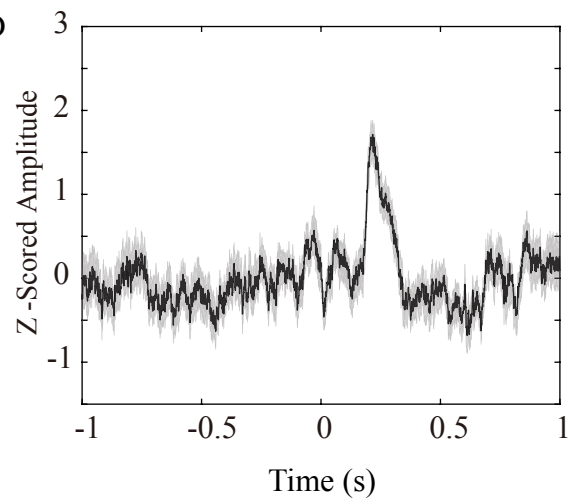

E

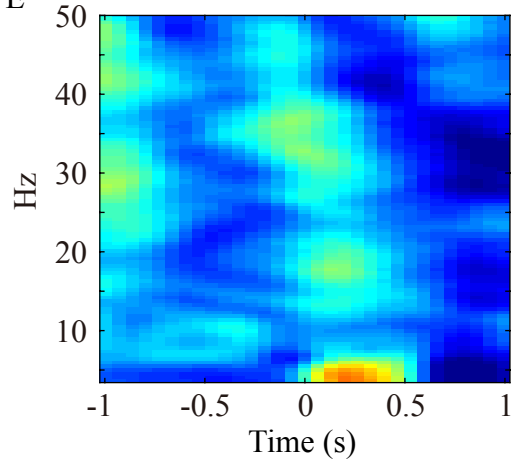

F

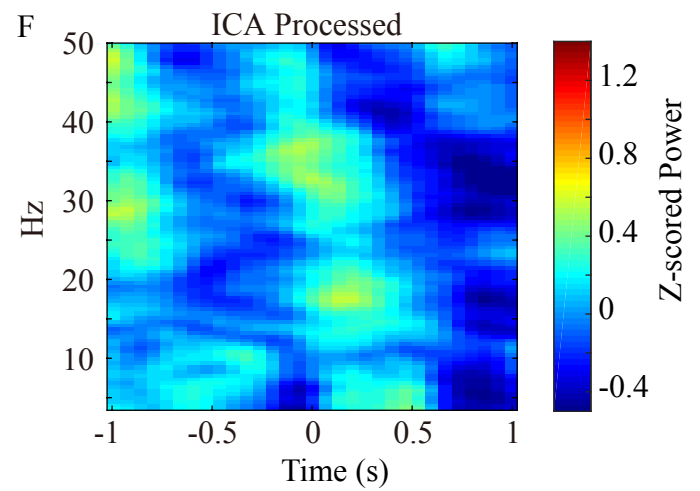

Supplement: Supplemental Information 5 — (A) and (D) are averaged LFP traces. (B) and (E) are averaged spectrogram of raw LFPs. (C) and (F) are averaged spectrogram of ICA pre-processed LFPs. (A) to (C) are from Bird 5 else are from Bird 6. [file peerj-07-7937-s005.pdf]
